# Sub-nanometre resolution imaging of polymer–fullerene photovoltaic blends using energy-filtered scanning electron microscopy

**DOI:** 10.1038/ncomms7928

**Published:** 2015-04-24

**Authors:** Robert C. Masters, Andrew J. Pearson, Tom S. Glen, Fabian-Cyril Sasam, Letian Li, Maurizio Dapor, Athene M. Donald, David G. Lidzey, Cornelia Rodenburg

**Affiliations:** 1 Department of Materials Science and Engineering, University of Sheffield, Sir Robert Hadfield Building, Mappin Street, Sheffield S1 3JD, UK; 2 Department of Physics, University of Cambridge, Cavendish Laboratory, 19 JJ Thomson Avenue, Cambridge CB3 0HE, UK; 3 FEI Co. Europe NanoPort, Achtseweg Noord 5, Eindhoven, 5651 GG, The Netherlands; 4 European Centre for Theoretical Studies in Nuclear Physics and Related Areas (ECT*-FBK) and Trento Institute for Fundamental Physics and Applications (TIFPA-INFN), via Sommarive 18, Trento I-38123, Italy; 5 Department of Physics and Astronomy, University of Sheffield, Hicks Building, Hounsfield Road, Sheffield S3 7RH, UK

## Abstract

The resolution capability of the scanning electron microscope has increased immensely in recent years, and is now within the sub-nanometre range, at least for inorganic materials. An equivalent advance has not yet been achieved for imaging the morphologies of nanostructured organic materials, such as organic photovoltaic blends. Here we show that energy-selective secondary electron detection can be used to obtain high-contrast, material-specific images of an organic photovoltaic blend. We also find that we can differentiate mixed phases from pure material phases in our data. The lateral resolution demonstrated is twice that previously reported from secondary electron imaging. Our results suggest that our energy-filtered scanning electron microscopy approach will be able to make major inroads into the understanding of complex, nano-structured organic materials.

## Abstract (web summary)

Morphological characterization of organic photovoltaic active layers is restricted by the lack of accurate chemical mapping tools. Here, the authors demonstrate an energy-filtered scanning electron microscopy technique, which enables sub-nanometre resolution imaging of an organic photovoltaic blend.

## Graphical abstract

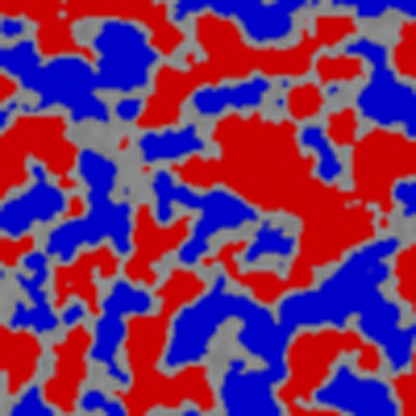

The scanning electron microscope (SEM) has undergone something of a minor revolution in recent years, to the point where it can now be truly considered at the cutting edge of imaging technology^1^. Sub-nanometre resolution is frequently observed in secondary electron (SE) images. Outstanding examples include the imaging of single uranium atoms^2^ and the topographical imaging of biological samples with 0.5 nm resolution^3^. However, the SEM remains uncompetitive as a tool for chemical mapping, especially with regards to nanostructured organic samples containing light elements. At present, acquiring chemical composition data in a SEM relies primarily on backscattered electron imaging or X-ray spectroscopy. Both these techniques have low spatial resolution in comparison to SE images, and struggle to distinguish between materials with similar elemental composition.

An excellent example of an area where the SEM has failed to make a significant impact is the characterization of nanoscale polymer blend morphology in organic photovoltaic (OPV)-active layers^4^. Here, a conjugated polymer and a fullerene are cast to form a bulk-heterojunction blend film exhibiting nanoscale phase separation^5^. The morphology of this blend is known to have a significant effect on the efficiency of an OPV device^4^, and as such characterizing these morphologies is hugely important to informing the development of more efficient devices. Despite its many benefits^6^, conventional SE imaging in the SEM is incapable of acquiring morphology data as the contrast between blend components is too low for nanometre-scale mapping^7^. Instead, energy-filtered transmission electron microscopy (EFTEM) is currently used for best-resolution imaging^8^,^9^,^10^,^11^,^12^. Here, blend maps are acquired by exploiting the electron energy-loss spectra of the blend components, to image in spectral windows in which the chemical contrast between the components is enhanced. Although lateral resolution of 1–2 nm is available from these techniques^10^, there remain obvious issues with the use of EFTEM on organic blend films: (i) the high level of knock-on damage relating to the large accelerating voltages inherent to transmission electron microscopy^13^ and (ii) the loss of depth resolution, as information is averaged over the entire specimen thickness. Although tomography can be employed to mitigate (ii), it exacerbates (i).

An alternative technique showing recent potential is energy-filtered scanning electron microscopy (EFSEM), based upon the energy spectroscopy of detected SE. Although such SE spectra are not widely known for exhibiting clear features related to sample chemistry, Joy *et al*^14^ have shown that they can be used for fingerprint identification of inorganic materials. Nonetheless, published applications of SE spectroscopy remain rare, because of the lack (at least until recently) of commercially available SEMs that enable systematic SE spectroscopy or high-resolution energy filtered SE imaging. EFSEM has, however, been previously employed for SE imaging of donor–acceptor junctions in silicon with improved dopant contrast^15^,^16^. Given that polymer–fullerene OPV blends are in essence donor–acceptor bulk heterojunctions, it is therefore a natural step forward to implement EFSEM to similarly improve material contrast in the characterization of organic semiconductor films.

The methodology for SE spectroscopy and EFSEM is described in detail elsewhere^17^. In brief, however, the sample is imaged using a through-lens detector (TLD), whereby SEs are extracted as they travel up the pole piece of the electron column and are deflected by a series of electrodes towards a scintillation detector mounted in the side of the column (the exact mechanism of deflection varies between SEMs). By altering the bias on one or more of these electrodes, a low-pass filter may be placed upon the detected SE. Where necessary, the electrode bias can be linked to a SE detection cutoff energy *E*_C_ by detector efficiency calculations, examples of which can be found in work published by Rodenburg *et al*.^15^ and Young *et al*.^18^.

Here, we apply EFSEM as a new method for obtaining a sub-nanometre chemical characterization of a *poly(3-hexylthiophene)*: *[6,6]-phenyl C61 butyric acid methyl ester* (P3HT:PCBM) blend. Our technique combines the depth resolution of SE images and reduced beam damage from a low-voltage electron beam with an unprecedented level of chemical contrast in our images through the use of the first SE spectra measured for OPV materials. We measure and compare the SE spectra of the individual blend components and identify a spectral window, in which one material is significantly more emissive than the other. By imaging the blend using only SEs in this window, we can boost the level of material contrast in our blend images. Sub-nanometre resolution is observed in our EFSEM images, a significant improvement over the previous best obtained by SE imaging^6^ or by EFTEM^10^. Importantly, we can identify clear regions of mixed phase in our blend images, showing the potential for mapping chemical composition based upon SE image contrast. This feature of EFSEM in particular will be of interest to materials scientists and biologists alike.

## Results

### SE spectra of pure films

**Figure 1:**
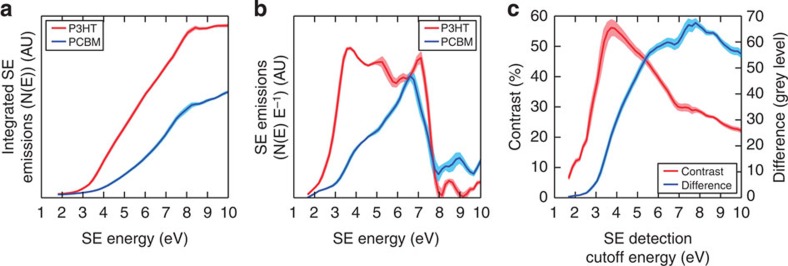
Measured SE spectra and contrast calculations. (**a**) The integrated SE spectra of P3HT and PCBM, averaged from multiple areas of pure samples. These plots are differentiated to give the SE spectra in **b**. (**c**) Plots both the raw brightness difference and contrast (calculated from the data in part **a** using equation (1)) between blend components as a function of *E*_C_. Shaded regions represent standard error on the mean from eight repeat measurements.

SE spectra of a sample are measured by taking a series of images while sweeping the bias on the TLD deflector electrode through a given range (see Methods section), and measuring how the detected SE signal changes as the bias (analogous here to the SE detection cutoff *E*_C_) is increased or decreased. Plotting the sample image grey level as a function of *E*_C_ gives the integrated SE spectrum of a sample as demonstrated in Fig. 1a, which we then differentiate to obtain the SE spectra in Fig. 1b.

The P3HT:PCBM blend was chosen as our test system because of it being a popular and well-studied active layer for OPV purposes, thus providing an excellent sample on which to demonstrate and validate a new imaging technique. It has admittedly been long surpassed in terms of OPV performance^5^, however, the EFSEM technique may be easily applied to other materials systems with similar results. We measured the SE spectra of pure P3HT and PCBM films individually, as presented in Fig. 1. Our spectra are reproducible for freshly cast films (the shaded regions present in the spectra indicate the average level of error between different films), and were measured with sequential forward and reverse bias sweeps to ensure the spectra are unchanged in both sweep directions (the exact experimental parameters are described in the Methods section). Importantly, we found that the SE signal from a sample is significantly more dependent on its material composition than the material state of its components. Details of our specific findings in this regard can be found in the Supplementary Information. As an example, however, the spectral shape of P3HT, consisting of three close but distinct peaks, is retained for samples subject to a thermal anneal, used to increase the crystallinity of a sample (Supplementary Fig. 1). In addition, integrated SE spectra measured for P3HT:PCBM blend samples lie, as expected, between those measured for pure P3HT and pure PCBM samples, implying that the use of the two components in a blend sample does not greatly affect the nature of the SE emissions from the two individual materials (Supplementary Fig. 2).

We believe that plasmon decay events are responsible for the characteristic shapes of the blend components' SE spectra in Fig. 1, or at least contribute to them. A detailed discussion of the shape of SE spectra is beyond the scope of this work, however, preliminary Monte Carlo modelling results (see Supplementary Fig. 3 and Supplementary Note 3) suggest that the shape reflects electron affinity and charge trapping. Previously it has been established that plasmons are significant contributors to SE emissions of energies 2–3 eV, based on measurements of SE emission from amorphous carbon films using an 80-keV primary electron beam published by Pijper and Kruit^19^.

### Contrast available from EFSEM

The clear spectral differences present between the SE emissions of P3HT and PCBM (Fig. 1a,b) enable the use of energy-filtered SE imaging to improve chemical contrast, defined here as *C*_*P3HT/PCBM*_. We use the integrated spectra in Fig. 1a, which represent the imaging grey value of the pure films as a function of *E*_c_, to predict *C*_*P3HT/PCBM*_ between pure P3HT and PCBM as a function of spectral cutoff energy, *E*_c_, using the SE contrast equation from Seiler^20^


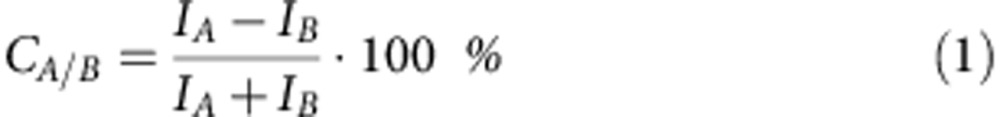


where *I*_*A*_,_*B*_ represents the measured grey value for P3HT and PCBM, respectively, at a given value of *E*_C_. The results of these calculations are shown in Fig. 1c, and predict the contrast between the blend components as a function of *E*_C_.

To obtain EFSEM images with high contrast and resolution, the choice of *E*_C_ used for imaging, based upon analysis of the SE spectra in Fig. 1, is critical. Owing to the lack of precedent in this field, our choice is based on the appropriate optimization of material contrast and signal-to-noise ratio (SNR). Using the contrast plotted as a function of *E*_c_ (Fig. 1c; along with raw SE intensity difference), we observe a clear peak in blend contrast at *E*_c_*∼*4 eV with *C*_*P3HT/PCBM*_ (4 eV)=(56±3)%. However, we note that imaging a blend morphology while filtering out all SE with *E*>4 eV would result in an unacceptably low SNR as data acquisition must be fast (that is, a short dwell time must be used) to minimize sample damage. The low signal available at this detection cutoff is self-evident from the integrated SE spectra in Fig. 1a, and we measured a SNR of 0.06 on a fresh blend surface using *E*_C_∼4 eV. We are unable to identify individual blend components using these parameters. Instead, we seek to improve the SNR by imaging using the cutoff point at which the numerical difference (*I*_*P3HT*_—*I*_*PCBM*_) between the blend component emissions is maximized (between 7.7 and 8 eV, as indicated by the ‘difference' plot in Fig. 1c). Here we still observe improved contrast of *C*_*P3HT/PCBM*_ (8 eV)=(29±1) %, and EFSEM images taken of fresh blend films with *E*_c_=8 V give a greatly improved SNR of 0.3.

**Figure 2:**
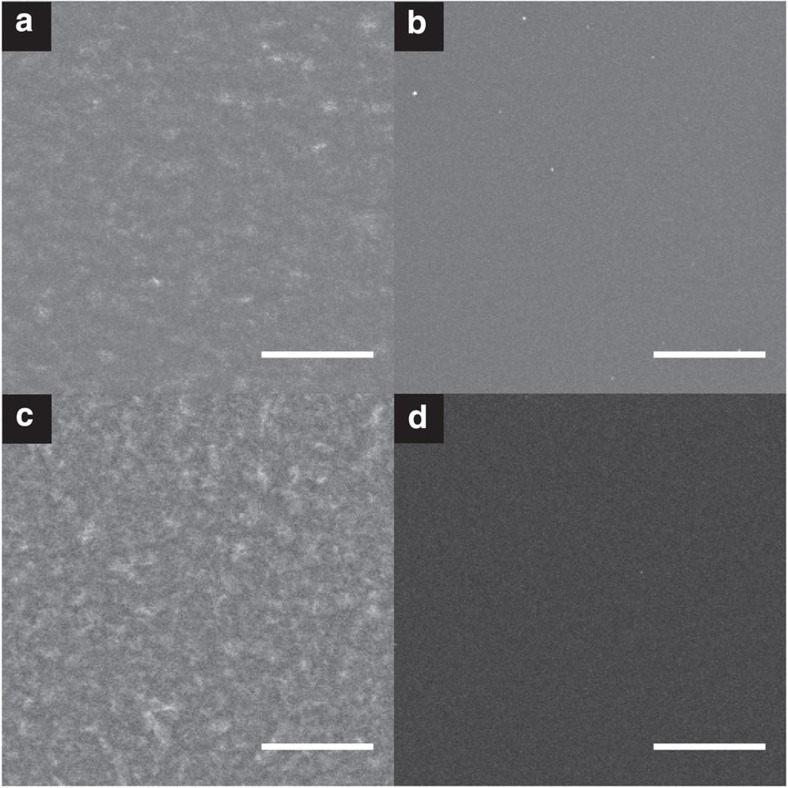
Low-magnification SE images of P3HT and PCBM blend pure films. (**a**,**b**) Unfiltered images of P3HT and PCBM, respectively, with (**c**, **d**) showing energy-filtered images of P3HT and PCBM pure films, using *E*_C_=8 eV. Scale bars represent 5 μm.

An example of the contrast improvement available from energy-filtered imaging is shown in Fig. 2. Here we compare SE images of pure P3HT and PCBM films, all taken using a high-resolution FEI Magellan FEGSEM with identical contrast and brightness settings. Figure 2a,b shows the two films as imaged under standard conditions with an Everhart-Thornley detector, whereas Fig. 2c,d shows the films imaged using the microscope's TLD with *E*_C_=8 eV. We note that the P3HT samples show some variation within the image, which we identify as being P3HT crystallites that are positioned close to or at the film surface; these appear with the use of high-resolution SEM equipment. Notably, the contrast appears more clearly in the energy-filtered image, possibly indicating that the contrast variations are linked to electron density changes (relating to crystallinity) at the surface rather than from topography. This effect has been observed in previous study^21^. To allow direct visual comparison of the improved material contrast available through energy-filtered imaging, the image brightness of Fig. 2c has been increased such that the mean grey level of Fig. 2c matches that of Fig 2a, with the brightness of Fig. 2d increased by an equal amount (thus, the grey level difference between Fig. 2c,d remains unchanged). Clearly, by comparing the contrast between Fig. 2c and d with that between Fig. 2a and b, the P3HT/PCBM imaging contrast has improved significantly using energy-filtered imaging. We find that this effect allows us to easily differentiate P3HT and PCBM in a high-resolution blend image.

### EFSEM images of blend films

**Figure 3:**
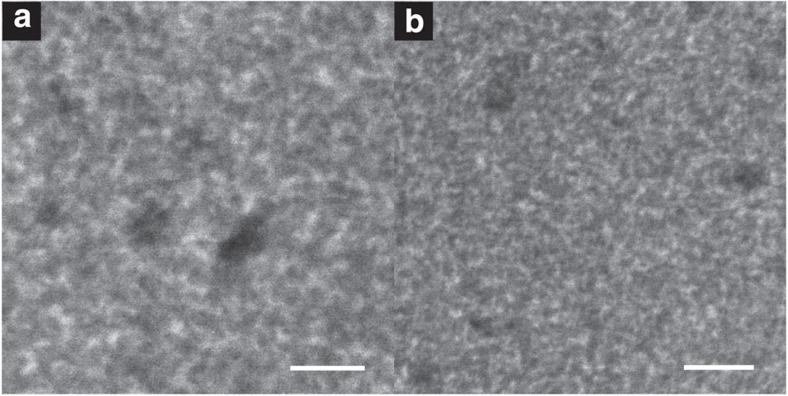
Overview of EFSEM results for P3HT:PCBM (1:0.8 wt%) blend. (**a**) Higher-magnification image (scale bar, 20 nm). (**b**) Lower-magnification image (scale bar, 30 nm). Spectroscopy of blend components suggests that brighter regions are P3HT-rich, darker are PCBM-rich. Clear mixed-phase regions are also visible.

Our blend film images (of a plasma cleaned film) are presented in Fig. 3a,b, covering different fields of view, hence using different electron doses (Fig. 3a used a dose of 9.36 × 10^−2^ C cm^−2^ and Fig. 3b used 3.66 × 10^−2^ C cm^−2^). Both show clear nanoscale variations in SE emission, which we attribute to the phase separation of the blend components; dark and bright areas can be identified as PCBM-rich and P3HT-rich regions, respectively, based on the contrast predicted in Fig. 1a. Directly visible in both images are areas with intermediate grey levels, which we identify as molecularly mixed-phase regions.

For an as-cast blend film surface imaged at *E*_C_=8 eV, we observed some extended nanostructures, however, no distinct phase separation could be observed on the surface of a fresh film (see Supplementary Fig. 4). We explain this as a result of a thin P3HT layer present at the surface of the film and obscuring the morphology beneath^22^. Similar observations were made from EFTEM analysis of P3HT:PCBM blend cross-sections published by Pfannmöller *et al*^10^. This polymer ‘skin' layer was removed using a plasma treatment, a technique previously shown to be effective in this regard^4^,^6^. The combination of EFSEM and plasma cleaning enabled us to collect the high-contrast, sub-nanometre resolution images presented in Fig. 3a,b.

**Figure 4:**
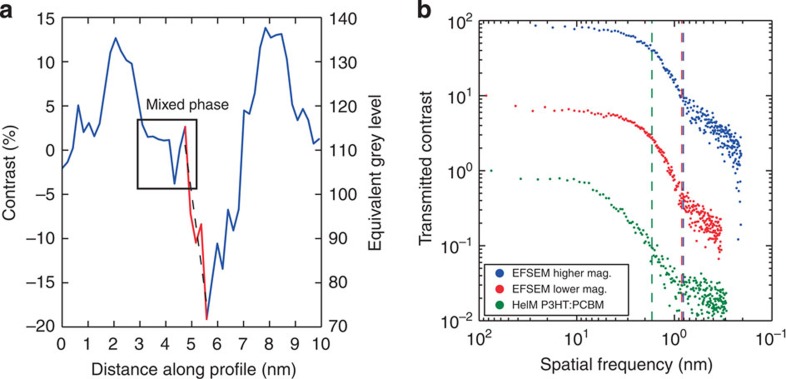
Analysis of EFSEM images in Fig. 3. (**a**) Line profile (1 pixel width) extracted from Fig. 3a, with a mixed phase region highlighted. The red part of the line profile shows an example concentration step used to calculate resolution. (**b**) A contrast transfer function for high-resolution images of P3HT:PCBM blends using both EFSEM and helium-ion microscope (HeIM). Each plot is offset by a factor of 10 for clarity. The noise-floor onset, correlating to the instrument's resolution limit, is highlighted for each plot. Mag., magnification.

We have quantified the level of contrast available in our blend images by taking 1-pixel-wide line profiles from the images, spanning P3HT-rich and PCBM-rich regions (an example is presented in Fig. 4a). Here we have once more used a percentage contrast scale based upon equation (1), where for clarity, the 0-point of contrast has been set around intermediate mixed-phase regions. From ten such line profiles, we estimate the average contrast between pure P3HT and pure PCBM regions in our images to be (35±4)%. This is almost double the material contrast seen when imaging without energy filtering (found to be (17±1)% for low-magnification data). Highlighted in Fig. 4a is an example region of intermediate ∼0% contrast seen to extend over >5 pixels, which as such is not an artefact of limited resolution. Mixed phase regions are expected in P3HT:PCBM blends, however, their nature and role in a functioning OPV device remains uncertain^23^,^24^, and therefore the ability to map these regions is hugely important in the context of OPV at present. To our knowledge, this is the first time such mixed phase regions have been imaged directly without the need to collect supplementary data (such as statistical analysis of electron energy-loss spectra spectra^10^,^11^) or to correct for factors such as sample thickness^25^.

### Spatial resolution of EFSEM images

We can determine the lateral resolution of our data using the definition of Kump and Diebold^26^, whereby resolution is defined as the distance over which the image brightness is seen to rise or fall between 16 and 84% of maximum brightness of a sharp concentration step. The line profile in Fig. 4a contains an example of such a concentration step (highlighted in the figure). We measure a resolution of (0.8±0.1)  nm for a step between pure PCBM and mixed phase, averaged from ten such line profiles. To show that this reflects the inherent resolution seen throughout our EFSEM data, we have calculated the contrast transfer function (CTF) of our images (as defined by Joy *et al*^27^,^28^). Here the onset of a noise floor sufficient to obscure the form of the CTF is defined as the resolution limit (see Fig. 4b). From Fig. 3a, we estimated the noise floor onset between spatial frequencies of 0.71-0.84 nm, and for Fig. 3b, we estimate it to lie between 0.63 and 0.81 nm. We can compare this to the CTF of helium-ion microscope images of similar plasma-cleaned P3HT:PCBM films^6^, where we observe a noise floor onset between 1.1 and 1.7 nm. Taking the upper estimates for these resolution limits, we can thus show that the lateral resolution available from our EFSEM technique is twice the previous best shown from SE imaging in the helium-ion microscope. We also note that this suggests that the lateral resolution available from EFSEM is approaching the dimensions of a single PCBM molecule^29^. However, the overall spatial resolution of our method depends on its depth resolution, which is that of the escape depth of the energy-filtered SE^20^. We believe this to be 2–2.7 nm based upon simulations of the inelastic mean free path of electrons in P3HT (see Supplementary Fig. 3) and from studies of amorphous carbon by Inada *et al*.^30^, however, the exact depth resolution may be dependent on the blend component and level of crystallinity^31^. The fact that we observe extended areas of intermediate contrast in perceived mixed phase regions indicates that the SE escape depth exceeds that of a PCBM molecule, and that our definition of mixed phase material will incorporate all material phases with a surface depth smaller than 2.7 nm. Nonetheless, this compares favourably to EFTEM studies of similar samples, where the lateral resolution is estimated as 1–2 nm (ref. 10) and the depth resolution is limited by the sample thickness.

### Consideration of sample damage

**Figure 5:**
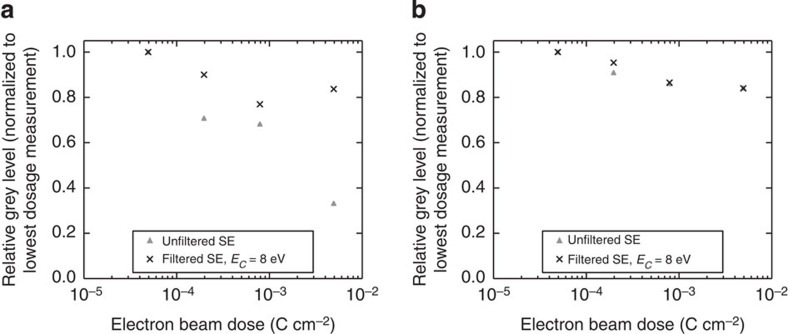
Relative grey level change resulting from electron beam irradiation. (**a**) Data for P3HT and (**b**) PCBM. Imaging grey level values are normalized to the grey level at lowest beam dose. Notably, P3HT retains its ‘brightness' at higher dose far more effectively using filtered imaging. Plots here show results for plasma-cleaned films, however, these results are unchanged with unprocessed films. Standard error on the mean for each data point is typically <3% based upon three repeat measurements.

Although one of the primary benefits of EFSEM in comparison to EFTEM is reduced sample damage, degradation resulting from primary electron beam irradiation and the plasma cleaning process remain a concern for this work. Electron beams in a transmission electron microscope or SEM are known to induce sample heating and sputtering in organic films, and will destroy the electronic properties of conjugated polymers in large enough doses^32^. In addition, surface contamination layers can quickly form as a result of chemical damage when organic samples are repeatedly scanned with electron beams^33^, although it has been shown that low-energy SE emissions are affected less by such formations^15^. These effects may change the nature of SE emissions from a material and affect the level of material contrast in our data. We have found that although imaging a pure film, increasing the electron dose (using a greater dwell time or magnification, for example) of an image irreversibly changes the grey level of the resulting image, which we assume correlates to changes in sample chemistry resulting from electron irradiation. The exact nature of this damage requires further investigation and will be addressed in future work; however, we can quantify the effect in Fig. 5.

Here we plot the change in imaging grey level relative to its value at × 5,000 magnification (a relatively small beam dose, ∼5 × 10^−5^ C cm^−2^) as the beam dose is increased by using larger magnifications. Most notably, the grey level of P3HT as imaged with unfiltered SE sees a significant drop in SE emission as the beam dose is increased to 0.005 C cm^−2^, to less than 40% of its emission at × 5,000 magnification. A grey level reduction at higher beam dosage is also observed with PCBM, albeit with a much smaller magnitude. However, when the films are imaged with energy filtering in place using *E*_C_=8 eV, this ‘darkening' effect is greatly diminished for P3HT. As images formed from low-energy SE appear to be affected less by electron beam irradiation, this may imply that the grey level change is at least partially a result of the formation of a modified surface layer. We nonetheless observe that SE emissions and material contrast are retained with energy-filtered SE (at least for *E*_C_=8 eV; both P3HT and PCBM see similar and small relative reductions in grey level at high magnification), whereas a significant negative effect is seen when using unfiltered SE. Thus, EFSEM allows for high-contrast imaging despite the apparent presence of beam damage.

We have also investigated the effect of plasma cleaning the samples by measuring the SE spectra of the pure materials after the same 6 min plasma cleaning process that our blend film was subject to. Although some changes were seen in the spectra after this plasma clean, the contrast between the materials using *E*_C_=8 eV was largely preserved. These spectra and related contrast calculations can be found in Supplementary Fig. 5. In addition, we refer to previous work that has measured the surface topography of similar blends by AFM following plasma cleaning in air; the length scale of the topography was found to be significantly larger than that of the contrast found in Fig. 3 (ref. 6). We are therefore confident that topographical variation is not contributing to the contrast in our high-magnification images.

### Morphology derived from image analysis

It is beyond the intended scope of this work to conduct an in-depth study of the relationship between blend processing parameters and morphology. However, to test the quality of our data and compare our results to similar experiments performed by other techniques, we have briefly characterized our blend images. The line profile in Fig. 4a demonstrates well-defined contrast levels for P3HT-rich, PCBM-rich and mixed-composition phases. Based upon ten representative line profiles, we have averaged the range of contrast levels for clear mixed-phase regions. We have subsequently calculated a contrast level for every pixel in our data; areas with contrast above the mixed-phase level have been deemed as P3HT-rich, areas with contrast below this are deemed PCBM-rich. We have found this to be an effective and reliable method, as can be seen from the results summarized in Table 1 for the two unprocessed energy-filtered images in Fig. 3a,b.

**Figure 6:**
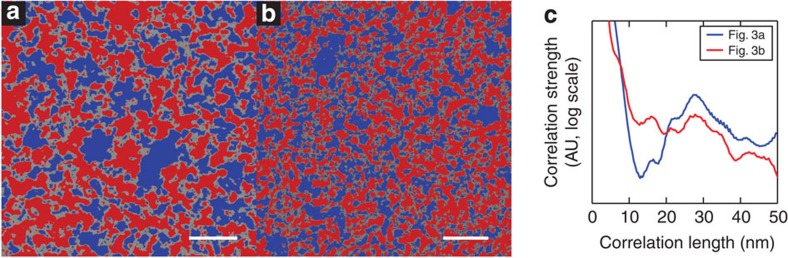
Summary of blend image characterization. (**a**,**b**) EFSEM images subject to FFT band-pass filter and thresholded to emphasize the imaged domain structure. Red areas correlate to those deemed to be P3HT-rich, and blue to those deemed to be PCBM-rich. The mixed phase is preserved in these images. **a** shows the same area as Fig. 3a (20 nm scale bar), and **b** the same area as Fig. 3b (30 nm scale bar). **c** shows radially averaged autocorrelation functions applied to Fig. 3a,b. Clear peaks in both functions are observed at ∼16 and ∼28 nm. Other, smaller peaks are also identified at longer correlation lengths.

Although this allows us to calculate phase distributions for a quantitative image characterization, we find a SNR of only 1.6 in our unprocessed images, whereas a SNR of 5 or better is recommended for this type of analysis^34^. Therefore, we employ a fast Fourier transform (FFT) band-pass filter to suppress noise in each image (specifically, structures of 3 pixels in size or smaller, corresponding to the noise floor level discussed in Fig. 4b). Although this affects the absolute contrast values in our data, we bypass this issue by considering the brightness of intermediate mixed phase regions in the FFT images, and thresholding around this level (see Methods section for more details). The threshold images obtained in this way are shown in Fig. 6, with the phase area calculations included in Table 1. In spite of the fact that Fig. 6a was taken at a total dose of ∼2.5x that of Fig. 6b, the percentage of mixed and pure phases is not changed within the uncertainty of our image analysis, and deviates by no more than 2%. This implies beam dosage is not significantly affecting the morphology data that we acquire.

We have also tested for average periodicity in our images. Radially averaged autocorrelation functions of the unprocessed images in Fig. 3a,b were calculated, with the results displayed in Fig. 6c. We find peaks at 16, 21 and 28 nm, with further, weaker correlations at greater lengths (this finds some agreement with power spectral density calculations made on EFTEM data by Pfannmöller *et al*.^10^). We find these length scales to be in the correct range for P3HT:PCBM blends^24^, and tentatively note that 28 nm corresponds to the separation between crystalline high *M*_W_ P3HT domains in pure samples^35^. Although this link may be purely coincidental, the fact that the morphology of a P3HT:PCBM blend is driven by the initial formation of P3HT crystallites^36^ means that we would likely expect the characteristic length scales of a P3HT:PCBM blend to reflect the properties of crystalline P3HT to a degree.

**Figure 7:**
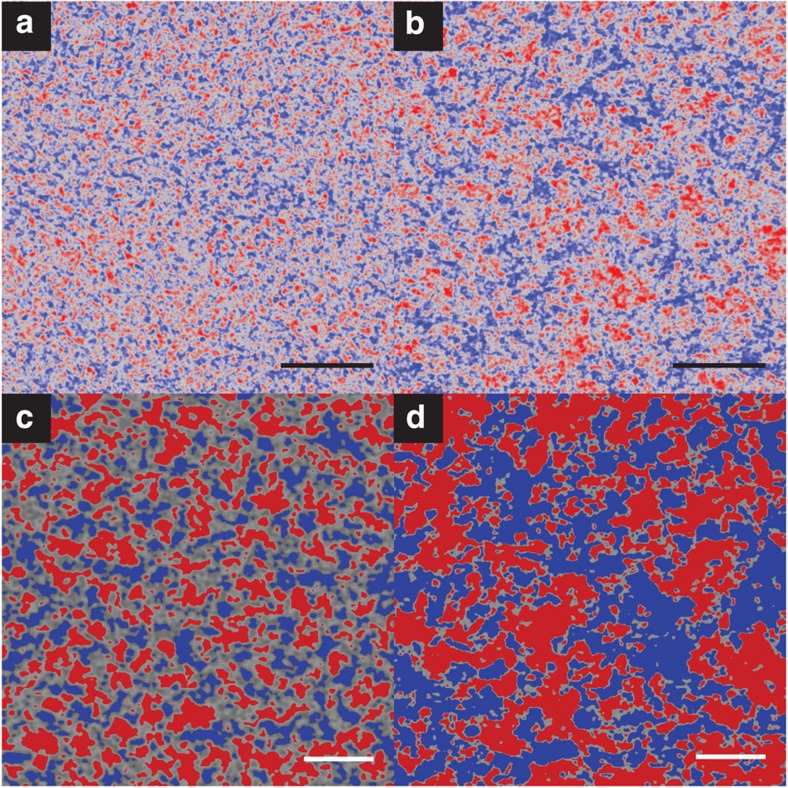
Blend images and characterization for samples subject to different thermal treatments. (**a**) The image data for an as-cast sample after a 2-pixel FFT band-pass filter to reduce noise, with (**b**) a comparable image for a blend subject to a 1-h over-anneal at 150 °C. Colour has been added to emphasize the phase structure visible in the data. Parts (**c**) and (**d**) show our thresholding attempts applied to higher-magnification data. Scale bars in parts (**a**) and (**b**) represent 100 nm, and in parts (**c**) and (**d**) represent 30 nm. For all parts, red areas correlate to P3HT-rich regions and blue to PCBM-rich regions.

Figure 7 demonstrates that EFSEM applied to the same blend materials but with different thermal treatments reveals the morphological changes resulting from thermal annealing. Figure 7a shows a sample not subject to any thermal anneal, while in Fig. 7b the blend has been deliberately over-annealed (for 60 min at 150 °C). This is in comparison to Figs 3 and 6, which reflect the morphology after a 10-min thermal anneal at 150°. Note the lower magnification of Fig. 7a,b, chosen to emphasize the larger-scale phase separation in Fig. 7b. In Fig. 7a, the imaged phase separation is on a shorter length scale, with large regions of intermediate grey level, which we allocate to mixed phase, separating the pure phases, whereas the over-annealed sample displayed in Fig. 7b shows larger pure phases of aggregated material and a diminished amount of mixed phase. Such clear changes in imaged morphology as a result of thermal treatments (with the expected trend^37^) offers further evidence that the contrast observed in our EFSEM images stems indeed from material variation.

We also applied our thresholding techniques to higher-magnification data for these samples, with the resulting thresholds displayed in Fig. 7c,d. A visual comparison of these images is sufficient to reveal the key differences between the samples, with Fig. 7c showing far greater mixed phase area and smaller pure phases in comparison to Fig. 7d. The phase area calculations for these thresholds can, however, be found in Supplementary Table 1.

## Discussion

With regards to the phase area calculations in Table 1, we note that 32% mixed phase was found by Pfannmöller *et al*. using EFTEM analysis of P3HT:PCBM blends^10^; however, due to the poor depth resolution in EFTEM (limited only by sample thickness) this value was in doubt. It is now supported by our measurements. In addition, we can compare our morphology data with results from bulk scattering studies of similar films by considering the concentration of PCBM present in the molecularly mixed phase. In the representative line profile in Fig. 4a, we observe that the contrast level of mixed phase lies approximately halfway between that of the pure P3HT and pure PCBM phases. This implies that the mixed phase is composed of roughly equal parts P3HT and PCBM. Using this assumption, we find that ∼14–18% of the blend volume consists of PCBM in mixed phase form. This figure finds good agreement with studies of similar films using small-angle X-ray scattering by Parnell *et al,*^38^, which suggest this figure to be 13%, and small-angle neutron scattering by Kiel *et al*.^39^, which suggest it to be 16%. Although it is known that many parameters can affect the precise morphology of any given P3HT:PCBM blend, these values are from largely comparable blends to the one presented in this work (in that they have been processed for optimal OPV performance), and use similar P3HT *Mw* and regioregularity where stated. This is an important correlation; although our results involve some assumptions, our data agree with scattering data reliant on a completely different and unrelated set of assumptions.

Data based upon averaged periodicity data are powerful, and the results of small-angle neutron scattering or X-ray scattering experiments on OPV blends have previously provided an excellent insight in to the nature of OPV-active layers. However, we believe that over-reliance on morphology characterization based purely on the averaging of bulk properties may lead to premature conclusions, whereby the effects of local variations in morphology or the shape of domains, for example, may be overlooked^40^,^41^. Directly imaging the sample is the only way to obtain morphology information of this type, and the combination of high resolution with clear chemical contrast is required for a morphology image to be of use in this regard. We expect high-quality morphology maps may also be beneficial for theorists, for example, as an input for Monte Carlo simulations of OPV devices^42^. Here we have demonstrated a method that can fulfil this requirement, by providing high-resolution morphology data that enables reliable and meaningful thresholding techniques for blend characterization.

Our analysis is based on images that have been thresholded rationally, which has been made possible by the use of SE spectra to define contrast levels between the component materials. This approach eliminates the obstacles usually encountered in attempting quantitative SEM analysis, including the variations in contrast between SEMs with different detector designs^28^. The blend processing parameters used for the samples presented in Figs 3 and 6 were chosen because they are known to produce good OPV performance for these specific materials. We had found indications for the presence of a mixed phase in this blend from earlier work^6^, however, limited resolution prevented any meaningful quantification of it. The application of EFSEM has allowed us to build upon this by directly imaging mixed-phase material. In summary, we have demonstrated sub-nanometre resolution images of a P3HT:PCBM blend morphology, using an energy-filtered SEM technique that exploits spectral differences in the SE emissions of the blend components. In addition to providing imaging resolution superior to that obtainable using competing techniques, EFSEM data are two-dimensional with few projection issues, and can be performed on wide sample areas with short (<1 min) acquisition times. The resolution and chemical contrast in our data have enabled a detailed characterization of the imaged morphology, using which we have demonstrated a powerful new technique for facilitating chemical mapping on a nanometre scale.

We hope that the image data presented here will boost interest in coincidence spectroscopy carried out at lower *E*_0_, in order to exploit EFSEM fully as an alternative to EFTEM. EFSEM can bypass the limitations of projection^43^ because of the small escape depth of SE, and also uses significantly reduced probe energy. As EFTEM is widely used in many materials science applications^44^ and is showing promise with biological samples^45^, we expect that many fields beyond the OPV community could benefit from the application of EFSEM in its stead.

## Methods

### Sample preparation

Polymer films were prepared by spin coating from solution on to silicon substrates. The substrates were cleaned in isopropanol before being plasma-cleaned in air for 15 min. P3HT (obtained from Ossila Ltd., brand Merck SP001 with 94.2% regioregularity and *M*_w_=54,200 Da) and PCBM (purchased from Solenne BV) were dissolved separately in chlorobenzene to make 25 mg ml^−1^ solutions, and heated to 70 °C overnight to aid dissolution. The solutions were mixed in a 1:0.8 (P3HT:PCBM) ratio by wt% to form the blend solution. All solutions (pure and blend) were spin-coated on to the silicon substrates at 1,500 r.p.m. in nitrogen atmosphere for 40 s to make the films. The P3HT:PCBM film for Figs 3 and 6 was thermally annealed at 150 °C for 10 min in accordance with standard practice for making efficient OPV morphologies. Samples for the images in Fig. 7 were either not thermally annealed at all (Fig. 7a), or annealed for 60 min at 150 °C (Fig. 7b). The sample substrates were attached to standard aluminium SEM stubs using conductive silver DAG paint acquired from Agar Scientific.

### Measurement of SE spectra

SE spectra were measured using a FEI Sirion FEGSEM with XL-30 tube assembly. Pure-film P3HT and PCBM samples were imaged using a 1-kV primary beam at 3 mm working distance, with SE collected using the immersion-lens TLD. Energy filtering of SE was performed by changing the TLD deflector electrode bias, *D*, while using a TLD tube bias of 250 V (ref. 17). *D* was correlated to the cutoff energy for SE detection using detector efficiency calculations published by Rodenburg *et al*. for an identical tube assembly using our imaging settings^15^. For a given value of *D,* we took the SE detection cutoff to be the energy at which SE detection efficiency drops below 30%. *D* was swept from 5 to 25 V in 0.5 V steps with a low-magnification (∼ × 2,500) image taken at each step. Using ImageJ, the grey level of eight 128 × 128-pixel regions across the image was averaged and plotted as a function of the SE energy, and this plot differentiated using OriginPro 9.0 software to produce the final SE spectra.

### Energy-filtered SE imaging

The P3HT:PCBM blend films for Figs 3 and 6 were imaged using the immersion-lens TLD of a FEI Helios NanoLab 660 FEGSEM, with the images in Fig. 7 taken at a later date using the same detector on a FEI Helios NanoLab G3, access to both of which was kindly provided by FEI Co. for the purposes of this experiment. For all high-resolution EFSEM images, a primary beam energy of 2.8 kV was used with a working distance of 1 mm, 3 μs dwell time and tube bias of 140 V. The samples were plasma cleaned (with an air-plasma) inside the SEM chamber for 6 min to remove surface layers from the polymer film. Energy filtering of SE was performed by altering the bias on the TLD mirror electrode, *M*. Detector efficiency calculations for this SEM tube were again used to correlate the value of *M* to a corresponding SE energy cutoff, these were provided in private communication with FEI Co. We found that a SE detection cutoff energy of ∼8 eV could be achieved by using *M*=−6 V.

The low-magnification images of P3HT and PCBM films (Fig. 2) were acquired using a FEI Magellan FEGSEM, with identical electron optics to the FEI Helios used for high-resolution imaging. Parts a and b were imaged using an Everhart-Thornley detector, with identical beam and sample settings to those used for the high-resolution blend imaging. For the energy-filtered images (Fig. 2c,d), the TLD was used for imaging, using *M*=−6 V. The same contrast and brightness settings were used for all sample images to allow their direct comparison.

### Image post-processing and analysis

All image post-analyses were performed in ImageJ. Our resolution calculations were performed in part using SMART-J^46^, a SEM image characterization plugin for ImageJ created and distributed by David C. Joy of UTK in private communication. For the thresholding and analysis of relative phase area in raw data, pixel brightness was converted to a contrast scale using equation (1) with the zero-point set as the mid-point between the grey level maxima and minima averaged from ten line profiles. The contrast range equating to the mixed phase was also calculated based upon the line profiles by calculating and averaging the contrast range of conspicuous mixed phase regions. Pixel contrast values above and below this contrast range were taken to represent either pure P3HT or pure PCBM, respectively. Particle analysis algorithms in ImageJ were used to calculate phase area. For quantitative analysis of Fig. 6a,b, we used a FFT band-pass filter to smooth structures of 3 pixels in size or smaller. Clear areas of mixed phase were identified in these images and the histograms of these areas taken. The grey level range corresponding to the mean of these histograms±1 standard deviation either side was taken to represent mixed phase regions. Between 10 and 15 such areas were used for each image analysed, and their properties averaged. Pixels with grey levels above and below the mixed phase range were taken to represent pure P3HT and pure PCBM domains, respectively. This same technique was used to threshold the images of non-annealed and over-annealed samples displayed in Fig. 7. The relative lack of mixed phase material in the over-annealed sample made this difficult for thresholding Fig. 7d, with the thresholds shown representing a best-attempt approximation of the phase areas present in the image.

## Author contributions

R.C.M. wrote the manuscript; C.R. conceived the experiment; C.R., D.G.L. and A.M.D. supervised the project. Experimental work was performed by R.C.M., A.J.P., T.S.G., F.-C.S. and L.L. Techniques for the modelling of SE spectra were conceived and implemented by M.D. Data processing and analysis were undertaken by R.C.M., C.R. and M.D. Technical and conceptual advice was provided by D.G.L., A.M.D. and A.J.P.

## Additional information

**How to cite this article:** Masters, R.C. *et al*. Sub-nanometre resolution imaging of polymer:fullerene photovoltaic blends using energy-filtered scanning electron microscopy. *Nat. Commun.* 6:6928 doi: 10.1038/ncomms7928 (2015).

## Supplementary Material

Supplementary Information Supplementary Figures 1-5, Supplementary Table 1, Supplementary Notes 1-5 and Supplementary References

## Figures and Tables

**Table 1:** Results of morphology characterization from image thresholding.

	% PCBM in crystalline aggregate form	% P3HT in crystalline form	% Mixed phase
3a (169 × 169 nm^2^) raw image	28±3	35±5	36±8
3b (105 × 105 nm^2^) raw image	29±2	36±4	36±6
6a (169 × 169 nm^2^) after FFT	30±3	40±3	30±6
6b (105 × 105 nm^2^) after FFT	30±2	42±2	28±5

P3HT, poly(3-hexylthiophene); PCBM, [6,6]-phenyl C61 butyric acid methyl ester.

Data show the total phase area observed in the raw images shown in Fig. 3 and the noise-reduced images presented in Fig. 6. Errors represent the variation in phase area within one standard error on the mean thresholding levels, calculated from 10 to 15 mixed phase areas.
