# Supplementary material for: Sub-nanometre resolution imaging of polymer–fullerene photovoltaic blends using energy-filtered scanning electron microscopy
Source: Nat Commun. 2015 Apr 24;6:6928. doi: 10.1038/ncomms7928 (PMC4423221; doi:10.1038/ncomms7928)
Supplement: Supplementary Information — Supplementary Figures 1-5, Supplementary Table 1, Supplementary Notes 1-5 and Supplementary References [file ncomms7928-s1.pdf]

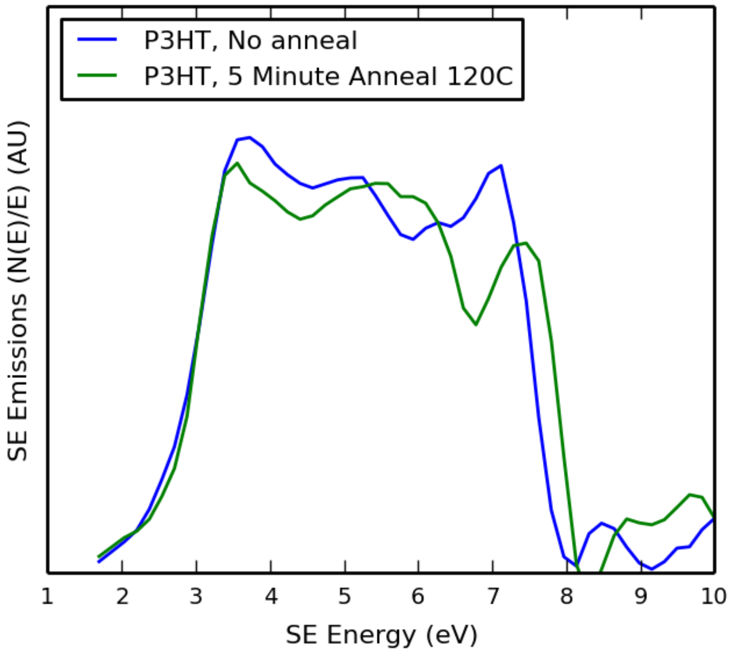

Supplementary Figure 1: Comparison of SE spectra from annealed and unannealed P3HT samples. See Supplementary Note 1 for discussion.

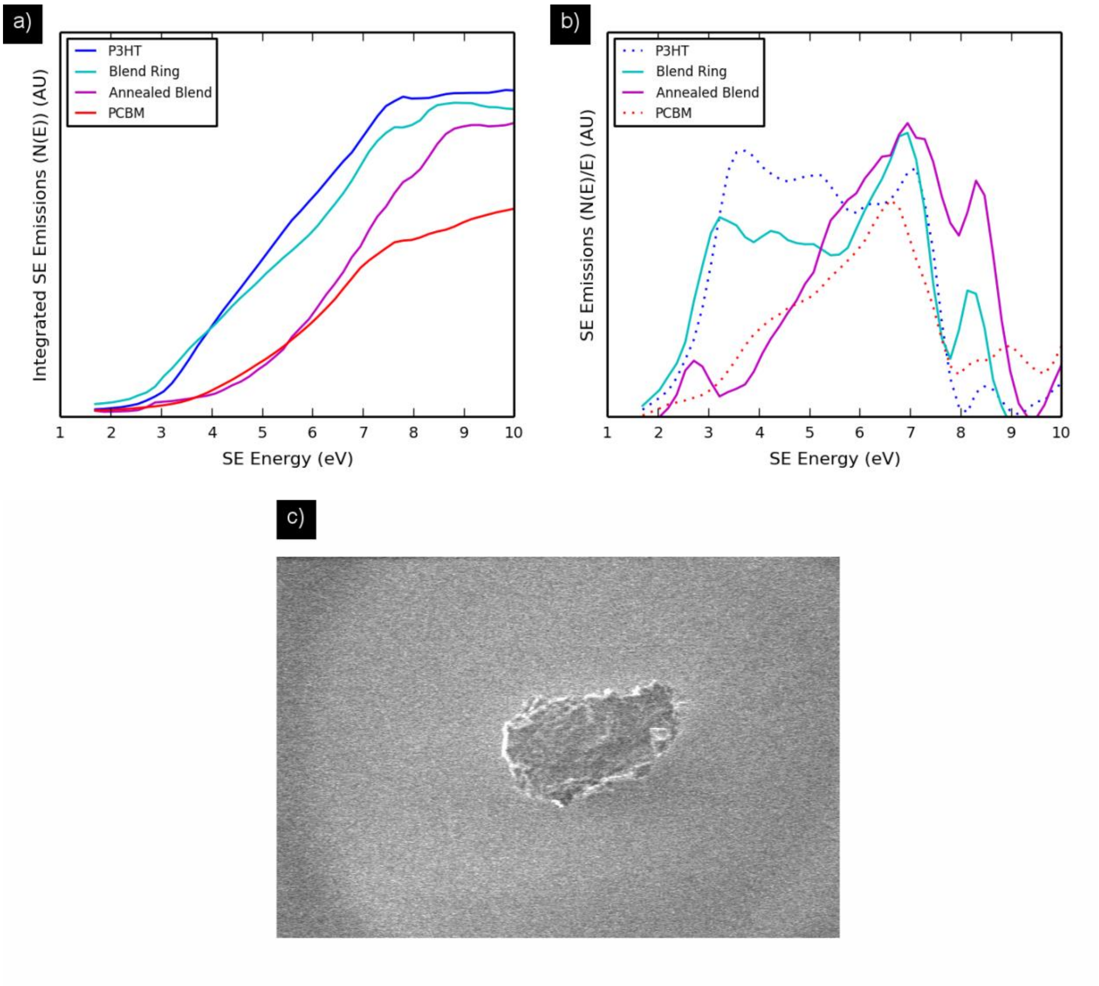

Supplementary Figure 2: SE spectra from blend films. a) and b) compare SE spectra from pure and blend films, in their integrated and differentiated forms respectively. c) is an example image from our SE spectra measurements, showing a bright P3HT-rich ‘ring’ around a film defect. See Supplementary Note 2 for discussion.

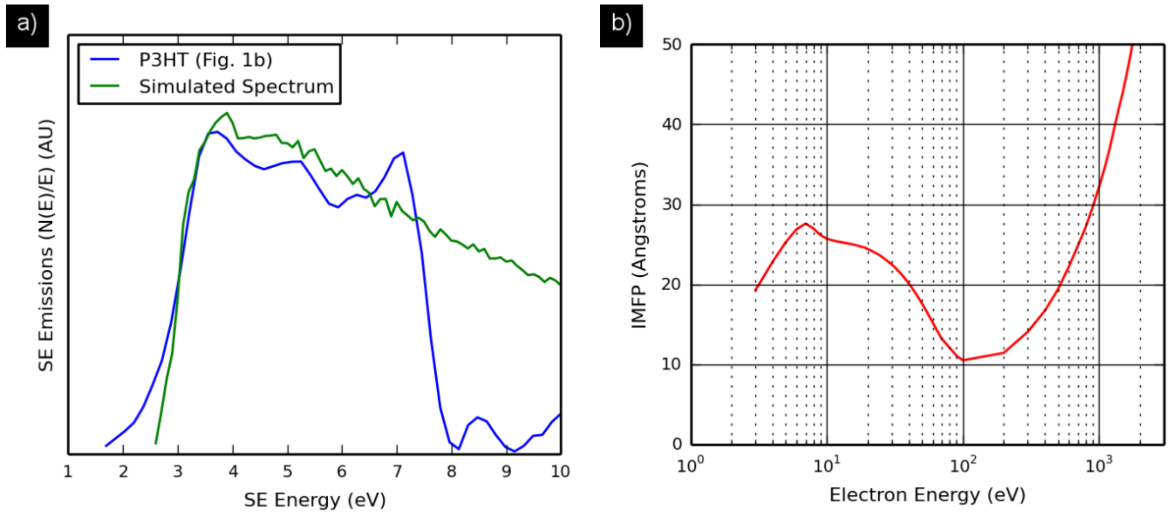

Supplementary Figure 3: Summary of SE spectra simulations. a) Comparison of P3HT SE spectrum from Fig. 1b and initial simulation results. b) Inelastic mean free path of electrons in P3HT as a function of electron energy, calculated in the process of simulating the SE spectra. See Supplementary Note 3 for a description of the simulations performed and a discussion of the results.

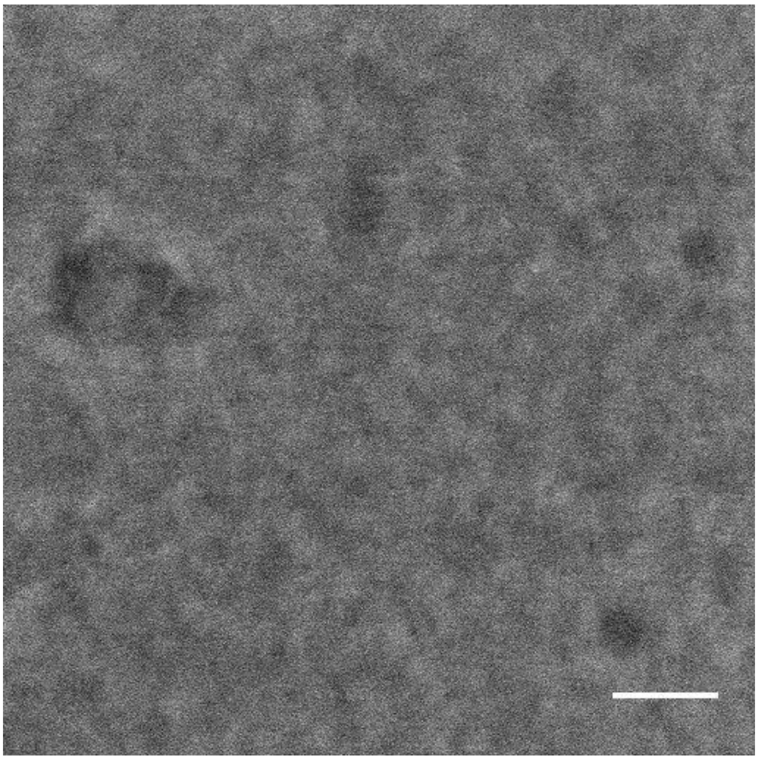

Supplementary Figure 4: EFSEM image (E_C = 8eV) of P3HT:PCBM blend prior to plasma cleaning. We observe that whilst some larger structures are visible in this image, the fine detail obtained in Fig. 3 is obscured by what is perceived to be a P3HT-rich ‘skin’ present at the surface of the film. The lack of detail available in this figure is our justification for the plasma cleaning of the blend film. The scale bar represents 30 nm.

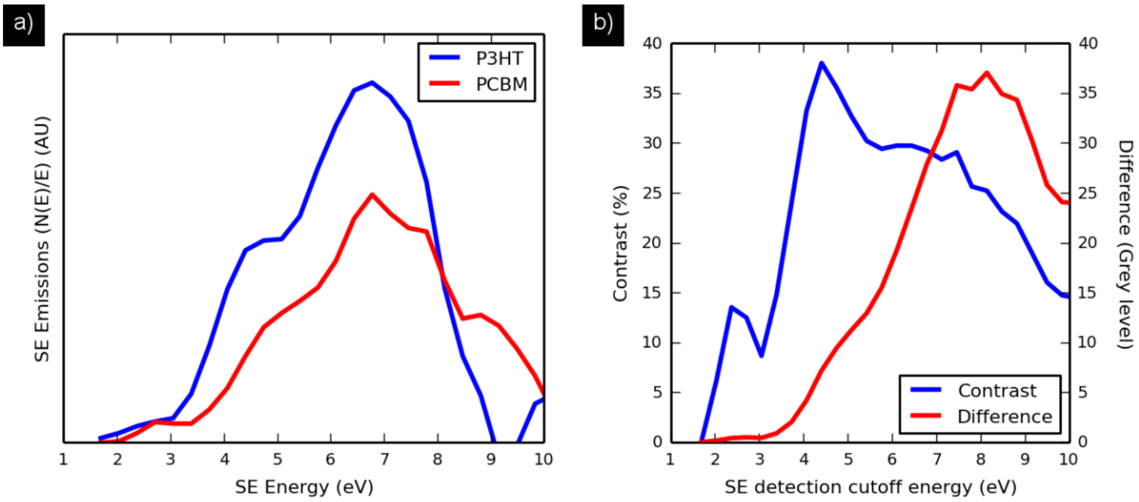

Supplementary Figure 5: SE spectra of plasma cleaned pure films. a) Differentiated SE spectra, b) Contrast and grey-level difference plots calculated from the integrated spectra (not shown). Contrast improvement is still available using E_C = 8 eV, see Supplementary Note 4.

**Sample**	**%P3HT**	**%PCBM**	**%Mixed Phase**
Fig. 7c, no thermal anneal	28.± 4	17 ± 3	54 ± 7
(Fig. 6a, 10 mins at 150°C)	*(40 ± 3)*	*(30 ± 3)*	*(28 ± 5)*
Fig. 7d, 60 mins at 150°C	42 ± 2	39 ± 2	18 ± 4

Supplementary Table 1: Phase area calculations for films subject to different thermal anneals. See Supplementary Note 5.

Supplementary Notes

Supplementary Note 1: In Supplementary Fig. 1, we address the notion of sample crystallinity having a potential effect on the nature of a sample's SE emissions. A pure P3HT sample was thermally annealed for 5 minutes at 120°C, in order to increase its level of crystallinity. Whilst the SE spectrum of this sample in Supplementary Fig. 1 is slightly changed with respect to that of the unannealed sample already presented in Fig. 1b in the primary manuscript, we observe the same basic spectrum shape and intensity between the annealed and unannealed samples. Thus we observe contrast available between different materials is mostly preserved despite varying levels of crystallinity within a sample, especially when employing energy-filtered imaging with E_C = 8 eV.

Supplementary Note 2: In Supplementary Fig. 2a, we plot the integrated spectra measured from P3HT:PCBM blend samples, along with the pure P3HT and PCBM samples presented in Fig. 1a of the main manuscript. Importantly, we find that the integrated SE spectrum for the blend sample (measured at low magnification, ~22 μm^2 area) lies directly between those measured for the pure materials at the 8eV cut-off point used for energy-filtered imaging. This fits our assertion that both materials are individually emitting SE with a similar energy spectrum to that found in pure samples. A second plot is also present in Supplementary Fig. 2a, originating from a ring around a PCBM-based defect found in the blend film. It is expected that local PCBM aggregates in to the defect, leaving a P3HT-rich area around it (see Supplementary Fig. 2c)[1]. We measured the SE spectrum of this 'ring' and found it to appear closer to that of a pure P3HT sample than the bulk film. This effect is more exaggerated in the differentiated SE spectra shown in Supplementary Fig. 2b. Once more, this emphasises that the SE spectrum measured from an area can be closely linked to the material composition of that area, largely independent of its material state. In the case of the P3HT-rich 'ring', its material state is undoubtedly affected by its presence in a blend film, specifically by the interspersion of remaining PCBM material within the 'ring'.

Supplementary Note 3: The SE spectrum for P3HT has been modelled in a preliminary form using Monte Carlo (MC) methods[2]. We consider the electron beam interaction cross-section, σ, to constitute the sum of all the electron scattering mechanisms experienced by the beam, such that:

$$\sigma = \sigma_{el} + \sigma_{inel} + \sigma_{ph} + \sigma_{pol} \qquad (1)$$

Where:

σ_{el} Is the elastic scattering cross-section due to the interaction between incident electrons and screened atomic nuclei, calculated using the relativistic partial wave expansion method (Mott cross-section)[3–5]

σ_{inel} Is the inelastic scattering cross-section due to the interaction between incident electrons and atomic electrons, calculated using Ritchie theory[6,7]

σ_{ph} Is the scattering cross-section due to electron-phonon interaction, calculated from Fröhlich theory[8,9]

σ_{pol} Is the scattering cross-section of the electron-polaron interaction, based upon work by Ganachaud and Mokrani[10].

Details of the precise Monte Carlo strategy used are covered in depth elsewhere[2], however the work is based primarily on the incorporation of P3HT EELS spectra from literature in to a model of the relationship between EELS and SE spectra. The results of one such simulation for the SE spectrum of P3HT are presented in Supplementary Fig. 3a, compared with the experimentally measured spectrum introduced in Fig. 1b in the main body of this work. We observe a reasonable fit of the simulated to experimental data, especially given that it relates to early work that will be greatly expanded upon in the future. Of particular note is the reproduction of the experimentally measured SE signal intensity at lower energies, as well as the presence of three peaks characteristic of the pure P3HT sample in the simulated data.

Inferring from the results of the simulation and the effects of changing various parameters in the model, we find that the lowest-energy of these three peaks can be identified as a result of the avalanche effect, and can be tuned by changing the electron affinity, χ, input in to the simulation. We find the best-fitting value for Fig. 1b to be χ = 2.5eV, which is lower than the literature value of 3.3 eV[11]. This probably reflects that the experimental sample was briefly exposed to air, likely forming a small oxidised layer on the surface and reducing the electron affinity of the sample. The second peak around 4.5 eV corresponds to the π-peak in the electron energy-loss function (ELF) for P3HT, whereas a third peak around 6 eV (somewhat obscured in Supplementary Fig. 5a) can be attributed to a joint contribution of the (π+σ) peak in the electron ELF, as well as the tail of the 'avalanche'. These correspond to peaks found in the experimentally observed spectra. Beyond ~7 eV our model begins to deviate, failing to reproduce a peak around 7 eV as well as the sharp drop-off observed in the experimental spectrum .This is likely a result of our preliminary data failing to take in to account some aspect of the material's interaction with the electron beam; in Supplementary Fig. 1 we have observed that a thermal anneal of a P3HT sample changes the intensity of the peak around ~7eV, therefore it is likely that the peak represents a sample property. We hope to address this in future work. However, we have established that the two highest-energy peaks represent very important aspects of the electronic properties of P3HT. We can therefore infer that SE spectra are indicative of material properties, with genuine variations in SE spectra between different materials.

In Supplementary Fig. 3b, the inelastic mean free path of electrons in P3HT as a function of electron energy is plotted. This data was acquired in the process of simulating the SE spectrum, and provides a good estimate of the escape depth of SE from P3HT whilst using energy-filtered imaging. At energies up to 8eV, we find this to be around 20-30 Å. This will be the limiting factor on the spatial resolution of our data.

Supplementary Note 4: Supplementary Fig. 4 shows the SE spectra of P3HT and PCBM films as measured after an equivalent 6-minute plasma clean process to that our P3HT:PCBM blend film was subject. We find that whilst the basic forms of both spectra change (the low-energy onset of P3HT emission is not as pronounced, and the peak emission of PCBM appears reduced), the basic contrast improvement afforded by energy-filtered imaging using E_C = 8 eV is preserved. This is emphasised by the projected contrast and difference plots, presented in Supplementary Fig. 4b, calculated from the integrated SE spectra measured for the plasma cleaned P3HT and PCBM films (not shown). Here we see there remains a clear contrast benefit for imaging using E_C = 8 eV.

Supplementary Note 5: Supplementary Table 1 displays the results of our phase area calculations for differently annealed samples. For the clearest results, the images in Fig. 7 were passed through a FFT bandpass filter before thresholding. The results of these phase area calculations should be directly comparable to those for Fig. 6b based upon the use of the same bandpass filter and magnification. Clearly, the mixed phase area present in our thresholded images decreases significantly for longer anneal times. We also observe the area fractions of pure phase material increasing with thermal annealing. These data match the expected trend for a thermally annealed sample; annealing above the glass transition temperature of the blend encourages greater phase separation of the materials, leading to larger, purer phases[12].

Supplementary References

1. Berriman, G. *et al.* Mapping chemical concentration in binary thin organic films via multi-wavelength scanning absorption microscopy (MWSAM). *Meas. Sci. Technol.* **25,** 095901 (2014).

2. Dapor, M. *Transport of Energetic Electrons in Solids*. *Springer Tracts Mod. Phys.* (Springer, 2014).

3. Mott, N. F. The Scattering of Fast Electrons by Atomic Nuclei. *Proc. R. Soc. A Math. Phys. Eng. Sci.* **124,** 425–442 (1929).

4. Dapor, M. Elastic scattering calculations for electrons and positrons in solid targets. *J. Appl. Phys.* **79,** 8406–8411 (1996).

5. Dapor, M. *Electron-Beam Interactions with Solids*. *Springer Tracts Mod. Phys.* (Springer, 2003).

6. Ritchie, R. H. Plasma Losses by Fast Electrons in Thin Films. *Phys. Rev.* **106,** 874–881 (1957).

7. Ritchie, R. H. & Howie, A. Electron excitation and the optical potential in electron microscopy. *Philos. Mag.* **36,** 463–481 (1977).

8. Fröhlich, H. Electrons in lattice fields. *Adv. Phys.* **3,** 325–361 (1954).

9. Llacer, J. & Garwin, E. L. Electron-phonon interaction in alkali halides. I. The transport of secondary electrons with energies between 0.25 and 7.5 eV. *J. Appl. Phys.* **40,** 2766–2775 (1969).

10. Ganachaud, J. P. & Mokrani, A. Theoretical study of the secondary electron emission of insulating targets. *Surf. Sci.* **334,** 329–341 (1995).

11. Miller, S. *et al.* Investigation of nanoscale morphological changes in organic photovoltaics during solvent vapor annealing. *J. Mater. Chem.* **18,** 306312 (2008).

12. Wang, T., Pearson, A. J., Lidzey, D. G. & Jones, R. A. L. Evolution of Structure, Optoelectronic Properties, and Device Performance of Polythiophene:Fullerene Solar Cells During Thermal Annealing. *Adv. Funct. Mater.* **21,** 1383–1390 (2011).
